# 

**Published:** 1892-02-27

**Authors:** 


**Notices of Births, Deaths, and Marriages,** are inserted in this column at a charge of 2s. 6d. for an announcement not exceeding four lines.

### NOTICE TO CORRESPONDENTS.

All MS., Letters, Books for Review, and other matters intended for the Editor should be addressed The Editor, The Lodge, Porchester Square, London, W.

The Editor cannot undertake to return rejected M.S., even when accompanied by stamped directed envelope.

**Notice to Advertisers and Subscribers.**

All Advertisements, Orders for Copies of The Hospital, and Business Communications should be addressed to The Publisher, *not to the Editor*, The Hospital, 140, Strand, W.C.

The Cost of Subscription, post free, is as follows:—Per week, 1½d.; per quarter, 1s. 8d.; per half-year, 3s. 3d.; per annum, 6s. 6d. Foreign:—Per annum, 8s. 8d.; payable, in all cases, in advance.

**"Burdett's Hospital Annual," 1891—1892.**

Third year of publication. 600 pp. Boards, gilt lettering. A most complete guide to British and Colonial Hospitals, Dispensaries, Nursing and Convalescent Institutions, and Asylums. Price 3s. 6d. Published by The Hospital (Limited), 140, Strand, W.C.

**NOTICE.—The Index for Vol. X. (ending September, 1891), is on sale at the Office of this Journal, price 2½d., post free.**

### GENERAL CHARITIES.

[This Column is devoted to an account of work of charitable institutions other than Medical Charities. The Editor will be glad to receive reports or news of work at 140, Strand. *Philanthropy* should be plainly written on the envelope.]

In a recent issue of this journal, a letter was inserted from Mrs. Hart, drawing attention to the existence of **THE DONEGAL INDUSTRIAL FUND.** This institution, which was founded in 1884 by Mrs. Ernest Hart for the revival and development of Irish home industries and the encouragement of hand-work, does not appear to be as widely known as we had hoped and believed it to be. The depot, which is at 43, Wigmore Street, W., helps no doubt to keep its existence before a certain number of the public, but, as a good number of people go about apparently with their eyes shut, we find that some people have often passed down the street in perfect ignorance of the existence of the shop where such excellent warm clothing can be purchased. Few, if any, homes are more widely known in Donegal than those of Mrs. Ernest Hart and Mrs. Power-Lalor, who have done so much at various times to alleviate the distress that is too common a dweller in the land. A touching story is told by Mrs. Hart of a visit she paid some few years ago to Donegal, when she met with an accident, being thrown out of a jaunting car. In conversation with some of the peasants the name of Mrs. Power-Lalor, who was her companion, was disclosed, whereupon one of the women said, seizing her hand, "And are *you* Mrs. Power-Lalor; you are not hurt, are you?" "No, I am not hurt at all," she replied. "Ah, no, you *couldn't* be hurt in Donegal," said the woman, "the children pray for you." This story speaks volumes for what can be done for the Irish by kindness. Recognising that these people are not the drunken and hopeless race so often represented, and that they only ask for work and the right to till the soil in peace, Mrs. Hart, with single-mindedness, set to work to enable these simple peasants to help themselves. This is a true form of charity which is worth every possible support. To give five guineas or ten guineas to an institution and have their names blazoned in the newspapers requires no self-denial, and argues no love for humanity or pity for its sufferings from those who can afford to give; but the devotion of time, labour, and love to going amongst a people and encouraging their industries demands many sacrifices, even though they may bring their reward. When Mrs. Hart first knew these people they were literally overwhelmed by distress. Her first efforts were devoted to stimulating industries which already existed and to teaching others that had been neglected or forgotten. This, however, was not enough, and it soon became evident that something more must be done if these industries were to be revived with lasting success. The work was useless unless a market could be found for it and definite orders could be given for the work. The key to the solution of this difficulty was the establishment of the depôt in Wigmore Street for the sale of Irish goods. The result of the stimulation and development of the cottage industries is to be seen not only in the increased material welfare of the peasants but in a revival of hope and intelligence which have had a marked influence in strengthening the character of the people. When starting this movement the chief aim was to give employment for hand-work and to provide technical teaching gratis. This has since been supplemented by the building of a steam-power factory at Gweedore, in the heart of the congested districts, where home-grown wool is worked up into tweeds, serges, flannels, blankets, &c., and by the establishment of a technical school and a carpenter's shop. It was found that the art of ordinary plain sewing and the making of garments was very little known among the women and girls in the district. This ignorance was to some extent accountable for from the fact that in some of the national schools there were no female teachers. Sewing classes were therefore started in the technical school and became very popular. The efforts to train Donegal boys in carpentering and wood carving was not made till four years ago; previous to this in many parishes there was not a single carpenter, and the spinning wheels and looms used by the weavers had to be carted over the mountains at great expense from places fifty or more miles away. The possession of the most ordinary articles of domestic furniture was therefore completely out of the question with the peasants of Donegal, a fact which only helped to accentuate the poverty of their surroundings. In order to start successfully their new industry it was necessary to bring two boys to London, where they remained two years and a quarter, and were thoroughly instructed in drawing, designing, wood carving, carpentry, building construction, and geometry. They were then sent back to Donegal in order to train Donegal boys as apprentices, and the carpentry and wood carving classes have been crowded with pupils eager to learn. Surely all this is work which should be encouraged by every possible means. There is one way in which everyone can help it on, and at the same time derive benefit to themselves from so doing, and that is by purchasing the products of these revived industries. The tweeds, linens, homespuns, under-linen, hosiery, laces, and embroideries, which are to be purchased at most moderate prices in Wigmore Street, have been awarded medals at various exhibitions in open competition with other goods. There is also, as Mrs. Hart pointed out in her letter, a stock of hand-made material, which, from being faulty only in design, is not up to the standard required by tailors, &c., and which would make excellent clothing for the poor.

### SCRAPS AND GLEANINGS.

THE Leathersellers' Company have given £10 10s. to the Irish Distressed Ladies' Fund.

THE workmen's subscriptions to the Workington Infirmary for the past year amounted to over £600.

THE Queen has sent a subscription of twenty guineas to the East London Hospital for Children at Shadwell.

THE Duke of Fife has consented to preside at the festival dinner of King's College Hospital, to be held on May 14th.

THE accounts of the Chelsea Hospital for the past year show that the cash receipts amount to £6,397. The balance on March 31st was £99 555.

THE *Court Circular* remarks that the Influenza scare has been for the most part purely imaginary and has been the result of alarmist paragraphs.

THE Lord Chancellor will preside at the annual dinner of the Hospital for Sick Children, Great Ormond Street, at the Hotel Metropole, on March 25th.

THE Richmond Hospital Sunday collections for 1891 realized £445; the Hospital Saturday collections £201 7s. 9d., and the total amount of box collections £440 6s. 10d.

MR. CHARLES H. WILSON, M.P., has consented to preside at the annual court of the Seamens' Hospital Society, to be held at Fishmongers' Hall on the 2nd of March.

DURING the year 1891 there were 347 intern and 7,593 extern patients at the Hospital for Sick Children, Belfast. The income for the year amounted to £966 4s. 9d., and the expenditure to £1,010 3s.

AT the annual meeting of the City Lying-in Hospital, Mr. Charles Gordon in the chair, it was reported that during the year 445 in-patients had been treated and 1,555 out-patients had also received the benefits of the charity.

THE Mercers' Company have contributed £21; the Armourers and Brasiers' 15 guineas; the Girdlers', 15 guineas; the Skinners', £10 guineas, and the Saddlers', £5 towards the new building fund of the Richmond Hospital.

THE report read at the annual meeting of the Metropolitan Hospital showed that the work had largely increased during the past 12 months. The receipts had decreased by £550, while the expenditure exhibited an increase of nearly £400.

MR. J. B. MARTIN, in presiding at the annual meeting of the Charing Cross Hospital, announced that the Hospital was now in a position to have a Convalescent Home, thanks to the munificent gift of Mr. J. Passmore Edwards.

THE annual report of the Essex and Colchester Hospital shows that the expenditure for the past year was £3,334 15s. 3d., and the year opens with the adverse balance of £149 3s. 5d. The admissions exceeded those of any year since the foundation of the hospital in 1828.

THE Governors of Torbay (Torquay) Hospital have decided to accept the munificent legacy of £7,000 bequeathed to the hospital under the will of the late Miss L. A. Warrington. It has long been wished to rebuild the hospital, and now this will be quite possible.

MR. CHARLES RAVENHILL, of the London Stock Exchange, has bequeathed to the London Hospital, St. Bartholomew's Hospital, the Brompton Hospital for Consumption, the Samaritan Free Hospital, £1,000 each, and £500 to the Stock Exchange Benevolent Fund.

THE Committee of the Royal Free Hospital, Gray's Inn Road, appeal for funds to carry out the restoration of the remaining portion of the structure; only half the amount needed (£20,000) has been received. By the kindness of friends, the greater portion of the old buildings have been replaced without an appeal to the public.

THE late Miss Charters has bequeathed £1,000 to the Samaritan Hospital for Women, Belfast. There was an increase of nearly 50 per cent in the amount of the public subscriptions for 1891 as compared with 1890, but there has been a decrease of £200 in the receipts from patients, which in previous years was the principal source of income.

AN interesting gathering took place recently in connection with the Convalescent Home for Men at Llanfairfechen. Miss Butlen, the Sister in charge of the home, held her first meeting at the Temperance Hall; Hanley, of former patients, about sixty men being present. A committee was formed, and it was decided to hold a concert as soon as possible on behalf of the institution.

THE total number of patients admitted to the St. John's Hospital for Skin Diseases and its convalescent branch last year was 5 923 being an increase of 1,080 over those of 1890. Of those 227 were admitted as in-patients; 5,659 received treatment as out-patients, and 56 were received into the convalescent branch at Finchley. The income of the year had amounted to £3,432, being an increase of £462 over that of the previous year.

THE promoters of the Chesterfield Hospital Penny Fund announce that £90 3s. 1½d. was collected in the boxes distributed in the town; other sums amounting to nearly £50 were received during the year. Relief had been granted in 329 cases. The Committee are anxious to have a ward set apart in connexion with the Hospital for the treatment of medical cases, so as to avoid the necessity of sending such cases to other hospitals.

THE Countess of Zetland, accompanied by the Marchioness of Drogheda, was present at a meeting held recently in Dublin for the purpose of establishing a consumption hospital in that city. A committee was formed and a subscription list opened. Miss Wynne, the originator of the movement, in announcing subscriptions to the amount of £3,000, added that unless £10,000 was subscribed within six months the committee would not go on with the undertaking.

THE Duke of Westminster presided at the annual meeting of the Chester Infirmary. The report stated that 841 in-patients, and 3,695 out-patients were treated. The expenditure (£6,043 4s. 6d.) exceeded the income by £654 15s. 6d. It was announced that £500 had been received from the Duke of Westminster, being a grant from the years' proceeds for viewing Eaton. During the past seven years the charity has benefited to the amount of £3,400 from this source alone.

### THE STUDENT OF MEDICINE.

[All communications for this Supplement should be addressed to The Editor of The Hospital, at 140, Strand, with the words, "Students' Column," written in the left hand top corner of the envelope.]

### APPOINTMENTS.

J. H. Bayley, M.B., C.M.Edin., appointed Assistant House Surgeon to the Northampton General Infirmary. W. H. Boazman, M.B., C.M.Edin., appointed Visiting Surgeon to the Chester General Infirmary, vice Dr. Wright. J. H. Bryant, M.D., B.S. Lond., appointed Medical Registrar and Demonstrator of Practical Medicine to Guy's Hospital. S. Bullivant, L.R.C.P.Lond., M.R.C.S., appointed Honorary Visiting House Surgeon to the Mansfield and Mansfield Woodhouse District Hospital. George J. Cressy, L.R.C.P., L.R.C.S.Irel., re-appointed Honorary Surgeon to the Mansfield and Mansfield Woodhouse District Hospital. Walter Dendy, M.B., C.M.Edin., appointed a Surgeon in Ordinary to the Bucks General Infirmary. J. Kingston Fowler, M.A., M.D.Cantab., L.R.C.P.Lond., M.R.C.S.Eng., appointed Physician to the Brompton Hospital for Consumption, vice F. T. Roberts, M.D., resigned. H. F. Knyvett, L.R.C.P., L.R.C.S. Edin., appointed House Surgeon to the Northampton General Infirmary, vice Durrant. J. M. Munro, M.B., C.M.Aberd., re-appointed Honorary Surgeon to the Mansfield and Mansfield Woodhouse District Hospital. George F. Phillpot, M.R.C.S.Eng., L.R.C.P.Edin., appointed a Surgeon in Ordinary to the Bucks General Infirmary. William Robertson, C.M. and M.D.Glas., appointed District and Dispensary Surgeon to the Perth County and City Royal Infirmary, vice W. Gowans, M.D.Edin., deceased.

### VACANCIES.

### Resident Medical Officers.

County Asylum, Rainhill, near Liverpool, Assistant Medical Officer; unmarried, and not more than 30 years of age.—Salary, which is progressive, £[illegible]00 per annum, with furnished apartments, board, washing, and attendance.

District Infirmary, Ashton-under-Lyne, Assistant House Surgeon and Dispenser.—Salary £50 per annum, with board and lodging.

London Temperance Hospital, Hampstead Road, N.W., House Surgeon, doubly qualified; appointment for six months.—Salary at the rate of 50 guineas per annum, with board, residence, and washing.

London Temperance Hospital, Hampstead Road, N.W., Junior House Surgeon, doubly qualified; appointment for six months.—Board, lodging, and washing provided, and an honorarium of five guineas at expiration of term.

Manchester Royal Infirmary, Assistant Medical Officer for the Monsall Fever Hospital; appointment for twelve months.—Salary £100 per annum, with board and residence.

Montrose Royal Lunatic Asylum, Junior Assistant Medical Officer.—Salary £80 per annum, with board, &c.

Newport and County Infirmary, Newport, Mon., House Surgeon, doubly qualified.—Salary £100 per annum, with board and residence.

Norfolk County Asylum, Thorpe, Norwich, Medical Officer, single, and under 30 years of age; appointment for five years.—Salary £16[illegible] per annum, with board, lodging, and washing.

Rotherham Hospital, Resident House Surgeon, doubly qualified, unmarried.—Salary £100 per annum, with board, furnished apartments, and washing.

Teignmouth, Dawlish, and Newton Infirmary, Dispensary, and Convalescent Home, House Surgeon, doubly qualified.—Salary £40 per annum, with board and lodging.

### Non-Resident Medical Officers.

Cancer Hospital, Free, Brompton, S.W., two Surgeons; must be F.R.C.S.Eng., and reside within the four miles radius.

County Down Infirmary, Surgeon. Application to the Registrar, co. Down Infirmary.

Dental Hospital of London, Leicester Square, two Assistant Dental Surgeons; must be Licentiates in Dental Surgery.

Dental Hospital of London and London School of Dental Surgery, Leicester Square, Demonstrator.—Honorarium, £50 per annum.

Hospital for Diseases of the Throat, Golden Square.—Vacancy on the Honorary Medical Staff.

London Temperance Hospital, Hampstead Road, N.W., Physician, doubly qualified.

London Temperance Hospital, Hampstead Road, N.W., Assistant Physician, doubly qualified.

Metropolitan Hospital, Kingsland Road, N.E., two Assistant Surgeons; must be F.R.C.S.Eng.

Middlesex Hospital, W., Anæsthetist.—Salary £100 per annum.

### THE STUDENT OF MEDICINE—(*continued*).

### NOTES FROM THE SCHOOLS.

#### University of Cambridge.

DEGREES.—At a congregation on January 21, the following degrees were conferred: M.D.: C. J. Whitby, B.A., M.B., Emmanuel. M.B. and B.C.: A. H. Godson, B.A., St. John's; A. Senior, B.A., Queen's: J. G. Gornall, B.A., St. Catharine's; W. C. Devereux, B.A., Selwyn.

At a congregation held on February 4th the Vice-Chancellor, presiding, the following degrees were conferred: M.B. and B.C.—J. Attlae, G. N. Edmonson, T. H. Evans, and L. G. Glover. St. John's; D. H. Attfield, Peterhouse; H. B. Grimsdale, Caius; E. B. Wrench, Downing; and W. Carling, non-collegiate.

#### Westminster Hospital.

Mr. M. Farrant read a paper on the "Colours of Animals." In the discussion which followed, Messrs. Yearsley, Winckworth, Bull, White, Swainson, Macleod, and Morris took part.

RUGBY FOOTBALL CLUB.—The above club held their annual smoking concert and supper on Friday evening. The attendance was large, and the affair was a marked success. Messrs. F. V. Denne and R. Nitch-Smith conducted the management.

GUTHRIE SOCIETY.—At the last meeting of the above society, Mr. Mark Farrant read a most interesting paper on "The Colour of Birds: Their Uses, Distinctions, &c., &c." A debate took place at the conclusion of the paper, and some points were well threshed out. At the last debate of this society, after several speakers had been heard and a division was called for, it was decided that "women should be debarred from entering the medical profession." Dr. Mills occupied the chair.

---

### PASS LIST.

#### Royal College of Surgeons, England.

The following gentlemen were admitted members of the College on February 11th: A. Adams, E. W. Adams, W. F. Adams, S. L. Archer, H. W. Armstrong, J. Atlee, C. Barker, J. A. Bell, H. G. Biddle, C. Billson, G. T. Bishop, A. S. Blackwell, H. B. Bolus, W. I. Boyes (M.B., Melbourne), C. B. Braithwaite, J. J. Browne, H. L. Brownlow, H. Burden, W. H. Cooke, A. T. Cooper, J. H. Day, W. H. Dolamore, M. R. P. Dorman, W. E. Drake, F. Ellis, R. B. Eccles, T. H. English, J. Evans, E. W. Everett, J. Fearnley, A. A. Fennings, H. Finley, R. C. Fraser, D. L. Freeland, L. G. Glover, C. H. Graham, J. Green, A. Greenhalgh, W. J. Gregerson (M.B., Melbourne), G. B. Griffiths, W. A. Haslam, A. G. Haydon, W. A. Hayes, W. A. Higgs, L. W. Hignett, R. Hopkin, J. W. Hutton, E. M. Iliewicz, G. P. Jerome, L. G. D. Jones, W. B. Jones, H. W. Kendall, C. A. Kitching, A. R. Lacey, S. H. Lee, T. Leicester, A. W. Lemarchand, F. Lewarne, H. W. Lewis, S. R. Lister, F. G. Lloyd, P. Lord, F. D. Lumley, C. R. Lunn, A. A. McAnally, A. A. McKinnon, *A. R. Mackinnon, A. W. McMichael, F. B. Marin, W. R. Matthews, J. Miller, A. G. Minshall, H. de R. Morgan, J. Morrison, G. E. Newby, S. R. Nicholls, G. Norman, A. E. Norris, W. W. Nuttall, J. E. S. Passmore, W. T. Pauling, A. S. Phillips, P. C. Phillips, J. S. Pickford, T. Pitt, F. C. Porter, P. C. Powrie, C. B. Prall, H. V. Prynne, C. H. D. Ralph, G. Ramsay, *M. A. Reid, W. J. Richardson, W. E. Roth, J. G. Rowell, F. B. Rutter, H. C. Sargent, H. D. Senior, *R. M. Smythe, *E. H. E. Stack, P. H. M. Star, E. J. Steegman, H. Thomas, W. Thomas, B. B. T. Thorne, J. G. Turner, C. P. Turner, C. H. C. Visick, E. C. B. Voisin, F. G. Wallace, T. Warner, H. G. Waters, D. J. G. Watkins, C. J. Weichert, A. L. Whitehead, C. H. Whiteford, T. R. Wiglesworth, H. S. Wild, H. B. Wilkinson, J. H. Williamson, C. G. R. Wood, H. E. Worthington, E. B. Wrench, J. T. R. Wylie.

Those marked with an asterisk (*) have passed the examinations for L.R.C.P.Lond.

#### Examining Board in England by the Royal Colleges of Physicians and of Surgeons.

#### Royal College of Physicians of Ireland.

At the monthly business meeting of the College, held on Friday, February 5th, 1892, the President admitted to the licences in Medicine and Midwifery the following candidates, who had been successful at the Final Professional Examination held in January under the Conjoint Scheme with the Royal College of Surgeons in Ireland: E. H. Beaman, H. Bouchier-Hayes, T. F. Dillon, J. J. M. Dowzer, M. Ferguson, F. W. Foott, E. F. Frazer, W. C. Hamilton, M. Keay, H. B. Ludlow, E. W. Lynch, J. M. Mangan, R. Moynahan, A. W. Power, G. H. Russell, J. G. Warren, R. J. White, G. T. Whyte, D. E. Williams. Mr. J. H. Elmes, who also passed the examination, being under age, was not admitted.

#### University of London.

PRELIMINARY SCIENTIFIC (M.B.) EXAMINATION: JANUARY 1892.

FIRST DIVISION.—H. Brown, B.A., V. E. Collins, G. T. Dickin, F. C. B. Gittings, W. Haigh, B.A., C. M. Hemmann, B.A., C. F. Hunter, J. A. McMichael, B.A., T. F. Rutter, S. R. Smith, A. G. Tolputt.

### THE STUDENT OF MEDICINE—(*continued*).

Second Division.—J. F. Baxter, H. Clifford, Octavia M. S. Lewin, F. H. Nimmo, H. P. Noble, A. E. Payne, R. O. Sibley, J. M. G. Swainson, A. J. Wernet, A. T. White.

Chemistry and Experimental Physics.—J. N. Bahadhurji,* E. C. Bailey, H. C. Barlow,* J. A. P. Barnes,* J. Blackwood, B.A.,* W. F. V. Bonney,* P. W. Brigstocke,* J. B. Christian, H. E. Corbin, A. B. Cridland, G. B. Crisp, T. B. Dakin, A. W. Dickson, M. Dixon,* Mary B. Douie,* H. R. Emms,* R. T. Fitz Hugh, F. E. Freemantle, A. B. Fry,* H. C. Harrison,* T. A. Hawkesworth,* Sindia E. Hickson,* Charlotte E. Hull,* G. K. Levick,* B. Lewitt,* C. D. Lindsey,* J. L. Maxwell, F. A. H. Michôd,* J. F. Northcott,* J. H. Phipps,* F. Pritchard,* C. H. Reissmann, C. H. D. Robbs, T. O'Neill Roe, C. Rundle,* A. G. Sargent, S. F. Smith,* J. H. Tallent,* W. E. Waymark,* H. E. Wise,* W. C. Wood.*

Biology.—W. B. Bell.* S. H. Berry,* E. C. Davenport,* C. W. Dimmock, C. E. Durrant,* W. N. East, J. H. Fillshill, H. N. Good,* J. J. C. Hamilton, G. B. Mason, R. Maxwell, C. Roberts, W. G. Savage,* F. M. Seal,* F. C. Shrubsall,* Margaret Smith, R Waterhouse,* W. B. H. Wood.

One Subject of the Examination (under former regulations). J. C. Harcourt (Biol.)* Private study.

* These Candidates have now completed the Examination.

### Society of Apothecaries of London.

The following candidates passed in:

Surgery.—W. H. Andrews, King's College; W. H. Cooke, St. George's Hospital; S. D. Gill, Birmingham (Queen's College); A. A. Macfarlane, St. Bartholomew's Hospital; J. N. Martin, St. Bartholomew's Hospital; C. S. Palmer, St. Bartholomew's Hospital; W. Robinson, Middlesex Hospital; J. H. F. Way, St. Thomas's Hospital.

Medicine, Forensic Medicine, and Midwifery.—J. D. Hessey, Middlesex Hospital; R. L. Romer, St. George's Hospital; H. G. White, St. George's Hospital.

Medicine and Forensic Medicine.—S. A. E. Griffiths, Middlesex Hospital.

Medicine and Midwifery.—R. S. Foulds, Charing Cross Hospital; J. P. Jones, London Hospital; C. Webb, St. George's Hospital; H. W. West, London Hospital.

Medicine.—C. R. Harper, Middlesex Hospital; A. G. Haydon, St. Bartholomew's Hospital; W. H. Waddington, Manchester (Owens College).

Forensic Medicine.—E. H. Bingley, St. Mary's Hospital; F. B. Lewis, London Hospital.

Midwifery.—W. C. Howle, Birmingham (Queen's College).

To Messrs. Bingley, Cooke, Griffiths, Harper, Haydon, Howle, Robinson, and White was granted the diploma of the Society entitling them to practice Medicine, Surgery, and Midwifery; enabling the holder to compete for medical appointments in the Army, Navy, and India Service, also for Poor-law appointments.

### ATHLETICS.

### Football.—Rugby.

Westminster Hospital v. Tooting College.—Won by Westminster, 19 points to nil.

Christ's Hospital v. Westminster Hospital (A Team) ended in a draw, both teams scoring seven points. The latter hospital played two men short.

Inter-Hospital Cup Ties (Second Round).

St. Thomas's Hospital beat Guy's Hospital by one try to nil.

St. Bartholomew's beat Westminster by three goals five tries (25 points) to nil.

February 13th.

St. Thomas's Hospital beat Harlequins by two goals and a try to one goal.

St. Thomas's Hospital beat Streatham Park by two tries to nil.

Saracens drew with London Hospital—no score.

Lennox (Second) beat Middlesex Hospital (Second) by one goal and four tries to one try.

Croydon (Second) beat St. Bartholomew's Hospital (Second) (R), at Croydon, by two goals (one penalty), or eight points, to nil.

Middlesex Wanderers (A) beat St. Mary's Hospital by one goal and two tries to nothing.

Chiswick (Reserves) beat Guy's Hospital (Second) by two goals (one dropped) and four tries to nil.

Inter-Hospital Challenge Cup (Semi-Final Round).

St. Thomas's Hospital v. Middlesex Hospital.—This tie was played yesterday at Richmond. The Middlesex forwards were completely outclassed by the St. Thomas's men, and victory rested with St. Thomas's Hospital by no fewer than four goals and six tries (32 points) to one try (two points).

Association.

Tunbridge Wells beat St. Mary's Hospital by one goal to nil.

Association Cup (Semi-Final Round).

St. Bartholomew's v. Guy's.—Won by St. Bartholomew's, after a good game, by four goals to love.

